# Immunological parameters and gene polymorphisms (*C-590T IL4, C-597A IL10*) in severe bronchial asthma in children from the Krasnoyarsk region, West Siberia

**DOI:** 10.3402/ijch.v72i0.21159

**Published:** 2013-08-05

**Authors:** Marina V. Smolnikova, Svetlana V. Smirnova, Maxim B. Freidin, Olga S. Tyutina

**Affiliations:** 1 Federal State Budget Institution “Research Institute for Medical Problems of the North”, Siberian Branch of Russian Academy of Medical Sciences, Krasnoyarsk, Russia; 2 Federal State Budget Institution “Research Institute for Medical Genetics”, Siberian Branch of Russian Academy of Medical Sciences, Tomsk, Russia

**Keywords:** asthma, cytokine, single-nucleotide polymorphism, IL-4, IL-10

## Abstract

**Background:**

Bronchial asthma is a common disease caused by interplay between multiple determinants, including genetic and immune variations.

**Objective:**

To investigate the main indices of humoral and cellular branches of immunity, features of cytokine regulation and cytokine genes in children with atopic bronchial asthma (BA) with different levels of disease control.

**Design:**

Fifty children with controlled BA (CBA) and 50 with uncontrolled BA (UBA) were analyzed. Mean age in the sample was 13.36±2.24 years. A control group of healthy children (n=50) was also studied. All individuals were Russians (Eastern Slavs) from the Krasnoyarsk Territory, West Siberia. Diagnoses, severity and level of disease control were defined according to the Global Initiative for Asthma (GINA) recommendations. The lymphocytes were counted in blood using fluorescent microscopy. Humoral branch indices and cytokine levels (IL-2, IL-4, IL-10 and TNF-α) in blood serum were measured by ELISA. Genotyping of single-nucleotide polymorphism (SNP) in −590 position of the *IL4* and −597 position of the *IL10* gene was performed by restriction fragment length analysis.

**Results:**

No statistically significant differences in total IgE and cytokines blood levels were found in CBA and UBA. However, significant differences between the groups were found for CD^3+^, CD^4+^ and CD^8+^ cell counts. The *T-590* allele of the *IL4* gene, which is responsible for an increased serum level of IL-4, showed a tendency to an association with UBA. A decreased level of IL-10 enhances control over BA, which proves its association with the allelic variant *A-597 IL10*.

**Conclusion:**

Our data show that children with UBA have higher counts of CD^3+^ cells and an increase of sub-population of CD^4+^-cells as well as higher levels of IgE, IL-4 and TNF-α in blood serum as compared to CBA. Polymorphisms of the *IL4* and *IL10* genes are associated with allergic inflammation in UBA.

Atopic bronchial asthma (BA) is the most common disease of the respiratory tract in children (1,2). It is characterized by repeated episodes of bronchial obstruction due to inflammation and bronchial hyperresponsiveness (2,3). Allergic inflammation is the common feature of BA and other atopic diseases.

Different cytokines control the development of allergic inflammation (4,5). Many of them show antagonistic relationships between one another. IL-4 is the main factor of regulation of allergic response through the control of immunoglobulin isotype switching in B-lymphocytes towards IgG and IgE expression (6). It also acts as a growth factor for T-lymphocytes and mast cells and is the key signal for the differentiation of CD^4+^-cells to T-helpers type 2 (7). These functional capabilities of IL-4 make it one of the most important cytokines for the development of BA (4,8,9). *IL4* gene is nearly 10 kb in length and contains four exons. Several single-nucleotide polymorphisms (SNPs) were revealed in the promoter region of the gene (*C-590T*, *C-285T* and *A-81G*). The allelic variant *T-590* is associated with higher promoter activity and increased production of IL-4 as compared to *C-590* allele (10). A polymorphism in the 3′-UTR region of *IL4* gene linked with the *C-590T* transition was shown to have prognostic significance regarding BA severity (10–12).

One of the key regulators of immune response is IL-10, which is synthesized by activated CD^4+^ and CD^8+^ T-lymphocytes, mast cells and activated monocytes (5,7). IL-10 inhibits the synthesis of a range of cytokines, produced by Th1 (IFN-γ, IL-2, TNF-β, IL-1, IL-6 and TNF-α), that leads to polarization of immune response to the Th2-profile (13–16). In allergic patients, there is a decrease of T-helpers producing IL-10 and low levels of IL-10 in serum (17,18). *IL10* gene is considered as a candidate gene for BA among the other innate immunity and immunoregulation genes (17,19). A number of SNPs in the gene were found to be associated with the production of IL-10 (*G-1082A*, *C-819T*, *C-597A*) (20). For instance, the *G* allele in the −*1082* position correlates with high production of IL-10 as compared to the alternative allele *A-1082*. The *A*-*597* allele is associated with the decrease of IL-10 production (19). The allelic variant *C-571* is associated with IgE hyperproduction and, as a rule, with a more severe course of BA (19). It has been shown that the *G-1082A* polymorphism of *IL10* along with the ATA haplotype in the promoter of *IL10* are associated with enhanced bronchial hyperresponsiveness, while the *T-819C* and *A-592C* polymorphisms as well as the ATA and ACC haplotypes are associated with the elevated levels of eosinophilic cationic protein in blood serum (21,22). Meta-analysis confirmed the association between the *G-1082A* and *C-597A* polymorphisms of *IL10* gene and atopic BA (23).

Stable elimination of BA symptoms is called control. Inflammatory biomarkers as well as pathophysiological signs can be used to trace the level of disease control. Recommendations on how to achieve BA control were developed and summarized in the Global Initiative for Asthma (GINA) document (2). The following goals need to be achieved: absence or minimal manifestation of symptoms; rare or lack of exacerbations and emergency calls; absence of physical activity limitations; normal lung function; minimal doses of medicines in case of emergency; no side effects of treatment. The goal of BA therapy is to achieve control over the disease by affecting inflammation pathways. Genetic polymorphisms associated with cytokine levels and lymphocytes counts can be useful biomarkers of individual features of patients which in turn can be taken into account for the adjustment of therapy regimes. Extreme ecological conditions of West Siberia result in the development of distinct adaptive functional and metabolic characteristics of people inhabiting the region. Immunological parameters are one of the most sensitive markers of such adaptations. In turn, this can cause specific patterns of BA morbidity, clinical course and therapeutic control in the West Siberia region. Therefore, we set out to investigate associations between BA control, immunological traits and polymorphisms of *IL4* and *IL10* genes in children from the city of Krasnoyarsk, West Siberia.

## Materials and methods

One hundred children from Krasnoyarsk with moderate to severe atopic BA with different disease control levels were studied. Diagnoses, severity and the level of disease control were defined according to recommendations of the report of working group GINA (updated 2008) (1). Before including a child in the study, we carried out a baseline clinical examination, which included the estimates of severity of clinical symptoms during the previous 3 months. For the enrolment to the study of the disease control level, the children along with their parents answered questions from the asthma control test (ACT) which is recommended by GINA. Until the beginning of 2007, ACT was available for adults and children older than 12 years only; however, in 2006, a younger children version has been released. An ACT score of>20 indicated controlled asthma, while an ACT score of <19 indicated uncontrolled asthma according to GINA criteria. The results of the test in the group of the controlled course was 22.45±2.6 points, in the uncontrolled course, it was 14.81±3.1 points (p<0.001).

The inclusion criteria for children to be part of the research were: diagnosis of atopic BA, moderate to severe disease course, absence of acute respiratory viral infection and other acute diseases at the time of the study, uninterrupted combinatory basic therapy in average or high therapeutic doses during the previous 3 months, Russian ethnic background in 3 generations. The criteria for healthy controls were: absence of the BA, absence of allergic diseases in family medical records, serum level of common IgE <100 IU/ml, Russian ethnic background in 3 generations. Our investigation was a case–control study.

After the results of clinical examination, 3 groups were formed: controlled BA (CBA) (n=50: 76% boys, 24% girls), uncontrolled BA (UBA) (n=50: 70% boys, 30% girls) and healthy control (n=50: 72% boys, 28% girls). Mean age of children with BA was 13.36±2.24 years (M±SD). Average duration of the disease in the CBA group was 6.12±3.29 years and in UBA 8.61±3.28 years. The control group of practically healthy children had an average age of 14.8±0.68 years. BA patients were enrolled in the study in the Krasnoyarsk Regional Children's Allergological Centre from 2009 to 2012. The healthy children were recruited from the secondary schools in the city of Krasnoyarsk. Informed consents for all participating children were obtained from their parents. The protocol of the study was approved by the Ethical Committee of the Research Institute for Medical Problems of the North.

Specific allergological examination included the collection of relevant medical history, skin-prick tests for domestic, epidermal and pollen allergens. Respiration function was measured by spirometry and pickflourometric test for reversibility of bronchial obstruction.

Peripheral blood was collected from fasting participants in the morning. Cytokine concentration in blood serum (IL-2, IL-4, IL-10 and TNF-α) was determined by ELISA (Vector-Best, Novosibirsk, Russia). DNA was isolated from 100-µl EDTA blood using Medigen DNA purification kit (MEDIGEN Laboratory, Novosibirsk, Russia).

Two polymorphisms in promoter regions of the cytokines genes were analyzed (*C-590T IL4* and *C-597A IL10*) in a subset of 31 children from CBA group and 33 children for UBA group. Genotyping was performed by polymerase chain reaction (PCR) followed by restriction endonuclease cleavage. PCR was carried out using primers described earlier (24–26).

Statistical analysis was performed using Statistica for Windows 6.0. Non-parametric Mann–Whitney U-test was applied to compare mean values of quantitative traits. Genotype frequencies were tested for deviation from Hardy-Weinberg equilibrium (HWE) by Fisher's exact test.

## Results

The study for parameters of immune status showed the statistically significant peculiarities of immune reactivity associated with the level of disease control (Table I). In CBA, increased levels of total lymphocyte counts, absolute counts of CD^3+^ cells and percentage of CD^8+^-lymphocytes in comparison with control were found. In the UBA group, as compared to the control, we revealed a statistically significant decrease in total lymphocyte counts, mature T-lymphocytes and CD4^+^ T-lymphocytes as well as a decrease in the immune regulatory index (CD^4+^/CD^8+^). However, the levels of CD^8+^ T-lymphocytes turned out to be evidently higher in UBA compared to the control. The differences between CBA and UBA were not pronounced, but were statistically significant.

**Table I:** Measures of parameters of cellular and humoral immunity in BA with different levels of control over the disease (M±SD)

Parameters	CBA (1) (n=50)	UBA (2) (n=50)	Control (3) (n=50)	p
Lymphocytes (%)	36.2±11.6	30.4±6.7	35.7±5.2	p_1,2_=0.023 p_1,__3_=0.0014 p_2,__3_=0.032 p_1,2,3_=0.07
CD^3+^ (%)	51.3±29.4	59.3±9.3	65.7±8.2	p_1_,_2_=0.028 p_1,__3_=0.025 p_2,__3_=0.028 p_1,2,3_=0.051
CD^3+^ (abs)	1467.1±258.0	837.0±3.9	1307.2±605.3	p_1,2_=0.014 p_1,3_=0.022 p_2,3_=0.0014 p_1,2,3_=0.0079
CD^4+^ (%)	20.6±12.6	25.1±0.03	37.3±5.9	p_1,2_=0.007 p_1,3_=0.0022 p_2,3_=0.00046 p_1,2,3_=0.003
CD^4+^ (abs)	461.3±37.9	220.6±11.8	807.5±27.5	p_1,2_=0.001 p_1,3_=0.014 p_2,3_=0.0003 p_1,2,3_=0.018
CD^8+^ (%)	38.4±9.7	36.1±7.6	25.5±4.9	p_1,2_=0.038 p_1,3_=0.048 p_2,3_=0.00023 p_1,2,3_=0.006
CD^8+^ (abs)	328.2±22.4	323.9±15.4	449.3±15.4	p_1,2_=0.0075 p_1,3_=0.024 p_2,3_=0.04 p_1,2,3_=0.004
CD^4+^/CD^8+^	0.66±0.24	0.75±0.4	1.44±0.34	p_1,2_=0.087 p_1,3_=0.0006 p_2,3_=0.0046 p_1,2,3_=0.0001
IgA (g/l)	1.39±0.67	1.78±0.99	1.98±1.96	p_1,2_=0.075 p_1,3_=0.048 p_2,3_=0.036 p_1,2,3_=0.04
IgM (g/l)	1.18±0.19	1.12±0.21	1.27±1.03	p_1,2_=0.117 p_1,3_=0.42 p_2,3_=0.36 p_1,2,3_=0.12
IgG (g/l)	14.22±4.05	13.72±5.12	11.57±5.21	p_1,2_=0.113 p_1,3_=0.06 p_2,3_=0.11 p_1,2,3_=0.062
IgE (IU/ml)	227.95±24.62	359.61±31.22	71.56±13.43	p_1,2_=0.057 p_1,3_<0.001 p_2,3_<0.001 p_1,2,3_=0.032

The analysis of the humoral branch of immunity showed a significant decrease of IgA levels in both groups of BA as compared with the control (Table I). In the CBA group, we found an increase of IgG concentration in blood serum in comparison with the control (p=0.06). The concentration of total IgE was statistically significantly higher in CBA and UBA groups compared to the control. Notably, the level of IgE was higher in UBA than in CBA (p=0.057).

The analysis of cytokine levels revealed a statistically significant increase of Th1-cytokines (IL-2, TNF-α) and Th2-cytokines (IL-4, IL-10) both in CBA and UBA groups as compared to the control (Table II). The Th2-cytokine levels in the UBA group were higher than in CBA. However, the differences were not statistically significant. In children with BA, the significant increase in TNF-α was observed. Also, the level of TNF-α in UBA was statistically significantly higher than in CBA (0.006).

**Table II:** Concentration of cytokines in blood serum in patients with BA with different levels of control over disease (Me (Q_25_–Q_75_))

No.	Group name	IL-2 (pg/ml)	IL-4 (pg/ml)	IL-10 (pg/ml)	TNF-α (pg/ml)
1	CBA (1) (n=50)	24.06 (7.96–38.43)	5.27 (2.85–7.82)	36.52 (20.51–48.72)	16.61 (15.29–29.52)
2	UBA (2) (n=50)	11.81 (8.53–30.57)	5.83 (3.79–14.83)	37.39 (21.73–42.63)	25.62 (18.62–31.48)
3	Control (3) (n=50)	5.23 (2.44–7.20)	2.45 (0.84–4.99)	15.89 (6.74–27.83)	6.63 (2.58–11.87)
4	P	p_1,2_=0.84 p_1,3_=0.006 p_2,3_=0.005	p_1,2_=0.48 p_1,3_=0.0019 p_2,3_<0.001	p_1,2_=0.98 p_1,3_<0.001 p_2,3_<0.001	p_1,2_=0.006 p_1,3_<0.001 p_2,3_<0.001

The study of *C-590T IL4* polymorphism showed the high incidence of the *CC* homozygote variant (56.2% in BA and 59.6% in control). The prevalence of the mutant variant genotype *TT* was minimal in the control group and more common in affected children (1.9 and 4.7%, respectively; p=0.015) (Table III).

**Table III:** Prevalence of the *-590 IL4* and *-597 IL10* genotypes in studied groups (%)

*IL4 C-590T*	Control (1) (n=50)	ABA (2) (n=64)	CBA (3) (n=31)	UBA (4) (n=33)	OR	CI	p
*CC* *CT* *TT*	59.6 38.5 1.9	56.2 39.1 4.7	61.3 35.5 3.2	51.5 42.4 6.1	OR_1,2_=0.87 OR_1,3_=1.07 OR_1,4_=1.19 OR_3,4_=1.49	CI_1,2_=0.39–1.9 CI_1,3_=0.39–2.9 CI_1,4_=0.76–1.5 CI_3,4_=0.49–4.5	p_1,2_=0.71 p_1,3_=0.83 p_1,4_=0.74 p_3,4_=0.43
*IL10 C-597A*					OR	CI	p
*CC* *CA* *AA*	73.1 26.9 0	62.5 29.7 7.8	61.3 29.0 9.7	63.6 30.3 6.1	OR_1,2_=1.88 OR_1,3_=2.05 OR_1,4_=1.73 OR_3,4_=0.84	CI_1,2_=0.89–4.2 CI_1,3_=0.85–4.1 CI_1,4_=0.86–4.2 CI_3,4_=0.34–2.1	p_1,2_=0.07 p_1,3_=0.08 p_1,4_=0.18 p_3,4_=0.68

The −597*AA* genotype of *IL10* gene was not found in the control group. Simultaneously, its prevalence in CBA was 9.7% (p=0.08). We showed the trend for correlation between the *A-597* allele variant with a decreased concentration of IL-10 in BA children. The prevalence of *A-597* variant of *IL10* in the BA group was higher than in the control group, though the difference did not reach statistical significance (p=0.074; OR: 1.88; CI: 0.89–4.1).

The levels of serum IL-4 in CBA and UBA groups as well as the level of IL-4 in the control group among the individuals with *−590CC* and −*590CT* genotypes of *IL4* gene were not significantly different. However, there was a trend towards an increase of the IL-4 serum levels in healthy carriers with −*590CT* genotype as compared to −*590CC* (3.4 and 2.0 pg/ml, respectively; p=0.07). No significant differences in serum levels of IL-10 among individuals with −*597CC* and −*597CA* genotypes of *IL10* were observed in the analyzed groups (Table IV).

**Table IV:** The level of the IL-4 and IL-10 in individuals with different genotypes of the *IL4* and *IL10* genes (pg/ml)

CBA (1) (n=31)		UBA (2) (n=33)		Control (3) (n=50)		
			
*IL4* *C-590T*	IL-4 Me (Q_25_–Q_75_)	*IL4* *C-590T*	IL-4 Me (Q_25_–Q_75_)	*IL4* *C-590T*	IL-4 Me (Q_25_–Q_75_)	p
*C/C* (n=19)	5.6 (2.8–7.9)	*C/C* (n=17)	5.1 (3.7–14.0)	*C/C* (n=39)	2.0 (1.3–2.1)	p_1,2_=0.018 p_1,3_=0.013 p_2,3_=0.082
*C/T* (n=12)	4.6 (3.3–6.1)	*C/T* (n=16)	8.3 (3.9–15.1)	*C/T* (n=11)	3.4 (2.4–4.4)	p_1.2_=0.056 p_1.3_=0.031 p_2.3_=0.24
	p=0.48		p=0.5		p=0.07	
*IL10 C-597A*	IL-10 Me (Q_25_–Q_75_)	*IL10* *C-597A*	IL-10 Me (Q_25_–Q_75_)	*IL10* *C-597A*	IL-10 Me (Q_25_–Q_75_)	
*C/C* (n= 19)	34.8 (21.3–51.3)	*C/C* (n=21)	39.1 (24.4–42.6)	*C/C* (n=42)	16.3 (9.6–20.7)	p_1.2_=0.0043 p_1.3_=0.003 p_2.3_=0.001
*C/A* (n=12)	39.6 (18.8–46.5)	*C/A* (n=12)	33.5 (19.8–39.6)	*C/A* (n=8)	13.9 (13.4–14.4)	p_1.2_=0.075 p_1.3_=0.019 p_2.3_=0.008
	p=0.66		p=0.17		p=0.64	

**Fig. 1:**
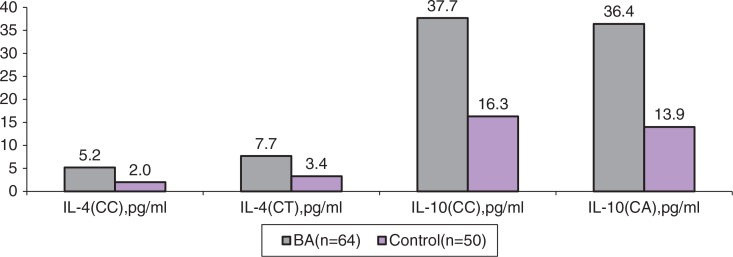
Cytokine level in blood serum (Me).

Median levels of IL-4 in BA children with *IL4* −*590CC* genotype were 5.2 pg/ml (Q_25_–Q_75_ range: 2.9–14.0), while in the control group they were 2.0 pg/ml (1.3–2.1) (p=0.0006) (Table V and Figure 1). In carriers with genotype *CT* in these groups, median levels of IL-4 were to 7.7 pg/ml (4.1–15.1) and 3.4 (2.4–4.4), respectively (p=0.012). Within the groups, in carriers with different genotypes (*CC* and *CT*), and also in CBA and UBA, the levels of IL-4 did not significantly differ. Median levels of serum IL-10 in BA children with *IL10 CC-597* genotype were 37.7 pg/ml (21.3–42.6), while in the control group they were 16.3 pg/ml (9.6–20.7) (p=0.05). In CBA and UBA groups, in carriers of *CC* genotype, we revealed statistically significant differences in IL-10 levels (p=0.0043). In BA carriers with *CA* genotype, the levels of IL-10 were 36.4 pg/ml (8.8–19.8), while in the control group they were 13.9 (13.4–14.4) (p=0.018).

**Table V:** The levels of the cytokines in individuals with different genotypes of the *IL4* and *IL10* genes (pg/ml)

BA (n=64)		Control (n=50)		p
*IL4 C-590T*	IL-4 Me (Q_25_–Q_75_)	*IL4* *C-590T*	IL-4 Me (Q_25_–Q_75_)	
*C/C* (n=36)	5.2 (2.9–14.0)	*C/C* (n=39)	2.0 (1.3–2.1)	0.0006
*C/T* (n=28)	7.7 (4.1–15.1)	*C/T* (n=11)	3.4 (2.4–4.4)	0.012
	p=0.065		p=0.07	
*IL10 C-597A*	IL-10 Me (Q_25_–Q_75_)	*IL10* *C-597A*	IL-10 Me (Q_25_–Q_75_)	
*C/C* (n=40)	37.7 (21.3–42.6)	*C/C* (n=42)	16.3 (9.6–20.7)	0.05
*C/A* (n=24)	36.4 (8.8–19.8)	*C/A* (n=8)	13.9 (13.4–14.4)	0.018
	p=0.28		p=0.65	

## Conclusion

The analysis of clinical, immunologic and genetic traits revealed specific features of uncontrolled course of BA in children from Krasnoyarsk region.

The parameters of immune status in children with UBA are characterized by T-lymphocytes deficiency, lower counts of CD^4+^ cells and lower CD^4+^/CD^8+^ ratio. The humoral branch of immunity expresses disimmunoglobulinemia, namely, the decrease in IgA production and over-production of IgE both in CBA and UBA groups as compared with the control. The revealed changes of humoral and cellular branches of immunity in children with BA with different levels of the disease control reflect specific features of atopy, such as IgE hyperproduction and T-lymphocyte deficiency, including Th-cells, as compared with the control group. It was shown that IgE deficiency due to persistent inflammation is most pronounced in cases of atopic BA with low disease control level.

The character of changes in the levels of cytokines in blood serum can be considered as a biomarker of uncontrolled course of atopic BA in children. This is based on the assumption that co-activation occurs in both Th1- and Th2-lymphocytes, as confirmed by high levels of cytokines TNF-α and IL-4.

It was shown that the *IL4 T-590* allele causes hyperproduction of IL-4 and tends to associate with an uncontrolled course of atopic BA due to IL-4 effects as the main modulator of allergic inflammation. The *IL10 A-597* allele is associated with low expression of IL-10 and influences the level of control over the course of atopic BA in children, preventing the deviation of immune response towards Th2 profile.

Under-diagnosis of BA among children is currently common worldwide leading to the development of severe uncontrolled forms of the disease course. Our study revealed clinical, immunological and genetic particularities of UBA in children from the Krasnoyarsk region; the data can be used as a reference to compare with children from other regions.
